# Phylogenetic Typology

**DOI:** 10.3389/fpsyg.2021.682132

**Published:** 2021-07-19

**Authors:** Gerhard Jäger, Johannes Wahle

**Affiliations:** Department of Linguistics, University of Tübingen, Tübingen, Germany

**Keywords:** typology, phylogenetics, Bayesian inference, word-order, language universals

## Abstract

In this article we propose a novel method to estimate the frequency distribution of linguistic variables while controlling for statistical non-independence due to shared ancestry. Unlike previous approaches, our technique uses all available data, from language families large and small as well as from isolates, while controlling for different degrees of relatedness on a continuous scale estimated from the data. Our approach involves three steps: First, distributions of phylogenies are inferred from lexical data. Second, these phylogenies are used as part of a statistical model to estimate transition rates between parameter states. Finally, the long-term equilibrium of the resulting Markov process is computed. As a case study, we investigate a series of potential word-order correlations across the languages of the world.

## 1. Introduction

One of the central research topics of linguistic typology concerns the distribution of structural properties across the languages of the world. Typologists are concerned with describing these distributions, understanding their determinants and identifying possible distributional dependencies between different linguistic features. Greenbergian language universals (Greenberg, 1963) provide prototypical examples of typological generalizations. Absolute universals^1^ describe the distribution of a single feature, while implicative universals^2^ state a dependency between different features. In subsequent work (such as Dryer, 1992), the quest for implicative universals was generalized to the study of *correlations* between features.

Validating such kind of findings requires statistical techniques, and the quest for suitable methods has been a research topic for the last 30 years. A major obstacle is the fact that languages are not independent samples—pairwise similarities may be the result of common descent or language contact. As the common statistical tests presuppose independence of samples, they are not readily applicable to cross-linguistic data.

One way to mitigate this effect—pioneered by Bell (1978), Dryer (1989), and Perkins (1989)—is to control for genealogy and areal effects when sampling. In the simplest case, only one language is sampled per genealogical unit, and statistical effects are applied to different macro-areas independently. More recent work often uses more sophisticated techniques such as repeated stratified random sampling (e.g., Blasi et al., 2016). Another approach currently gaining traction is the usage of (generalized) mixed-effects models (Breslow and Clayton, 1993), where genealogical units such as families or genera, as well as linguistic areas, are random effects see, e.g., Atkinson (2011), Bentz and Winter (2013), and Jaeger et al. (2011) for applications to typology.

In a seminal paper, Maslova (2000) proposes an entirely different conceptual take on the problems of typological generalizations and typological sampling. Briefly put, if languages of type A (e.g., nominative-accusative marking) are more frequent than languages of type B (e.g., ergative-absolutive marking), this may be due to three different reasons: (1) diachronic shifts B→A are more likely than shifts A→B; (2) proto-languages of type A diversified stronger than those of type B, and the daughter languages mostly preserve their ancestor's type, and (3) proto-world, or the proto-languages at relevant prehistoric population bottlenecks, happened to be of type A, and this asymmetry is maintained due to diachronic inertia. Only the first type of reason is potentially linguistically interesting and amenable to a cognitive or functional explanation. Reasons of category (2) or (3) reflect contingent accidents. Stratified sampling controls for biases due to (2), but it is hard to factor out (1) from (3) on the basis of synchronic data. Maslova suggests that the theory of Markov processes offers a principled solution. If it is possible to estimate the diachronic transition probabilities, and if one assumes that language change has the Markov property (i.e., is memoryless), one can compute the long-term equilibrium probability of this Markov process. This equilibrium distribution should be used as the basis to identify linguistically meaningful distributional universals.

Maslova and Nikitina (2007) make proposals how to implement this research program involving the systematic gauging of the distribution of the features in question within language families.

Bickel (2011, 2013) introduces the *Family Bias Theory* as a statistical technique to detect biased distributions of feature values across languages of different lineages while controlling for statistical non-independence. Briefly put, the method assesses the tendency for biased distributions within families on the basis of large families, and extrapolates the results to small families and isolates.

In this article we will propose an implementation of Maslova's program that makes use of algorithmic techniques from computational biology, especially the *phylogenetic comparative method*. A technically similar approach has been pursued by Dunn et al. (2011), where it was confined to individual language families. Here we will propose an extension of their method that uses data from several language families and isolates simultaneously. Unlike the above-mentioned approaches, our method makes use of the entire phylogenetic structures of language families including branch lengths—to be estimated from lexical data—, and it treats large families, small families, and isolates completely alike. Also, our method is formulated as a generative model in the statistical sense. This affords the usage of standard techniques from Bayesian statistics such as inferring posterior uncertainty of latent parameter values, predictive checks via simulations, and statistical model comparison.

The model will be exemplified with a study of the potential correlations between eight word-order features from the *World Atlas of Language Structure* (Dryer and Haspelmath, 2013) that were also used by Dunn et al. (2011).

## 2. Statistical Analysis

### 2.1. Continuous Time Markov Processes

Following Maslova (2000), we assume that the diachronic change of typological features follows a continuous time Markov process (abbreviated as CTMC, for continuous time Markov chain). Briefly put, this means that a language is always in one of finitely many different states. Spontaneous mutations can occur at any point in time. If a mutation occurs, the language switches to some other state according to some probability distribution. This process has the Markov property, i.e., the mutation probabilities—both the chance of a mutation occurring, and the probabilities of the mutation targets—only depend on the state the language is in, not on its history^3^.

Mathematically, the properties of such a process can be succinctly expressed in a single *n* × *n*-matrix *Q*, where *n* is the number of states. The entries along the diagonal are non-positive and all other entries non-negative. Each row sums up to 0. The waiting time until the next mutation, when being in state *i*, is an exponentially distributed random variable with rate parameter −*q*_*ii*_. If a mutation occurs while being in state *i*, the probability of a mutation *i* → *j* is proportional to *q*_*ij*_.

The probability of a language ending up in state *j* after a time interval *t* when being in state *i* at the beginning of the interval (regardless of the number and type of mutations happening during the interval) is *p*_*ij*_, where

$$P=e^{tQ}.$$

(The number *e* is the base of the natural logarithm). According to the theory of Markov processes^4^, if each state can be reached from each other state in a finite amount of time, there is a unique equilibrium distribution π. Regardless of the initial distribution, the probability of a language being in state *i* after time *t* converges to π_*i*_ when *t* grows to infinity. Also, the proportion of time a language spent in state *i* during a time interval *t* converges to π_*i*_ when *t* grows to infinity. According to Maslova, it is this equilibrium distribution π that affords linguistic insights and that therefore should be identified in distributional typology.

### 2.2. Phylogenetic Markov Chains

Different languages are not samples from independent runs of such a CTMC. Rather, their properties are correlated due to common descent, which can be represented by a *language phylogeny*. A phylogeny is a family tree of related languages with the common ancestor at the root and the extant (or documented) languages at the leaves. Unlike the family trees used in classical historical linguistics, branches of a phylogeny have a length, i.e., a positive real number that is proportional to the time interval the branch represents. According to the model used here, when a language splits into two daughter languages, those initially have the same state but then evolve independently according to the same CTMC. This is schematically illustrated in Figure 1.

**Figure 1:**
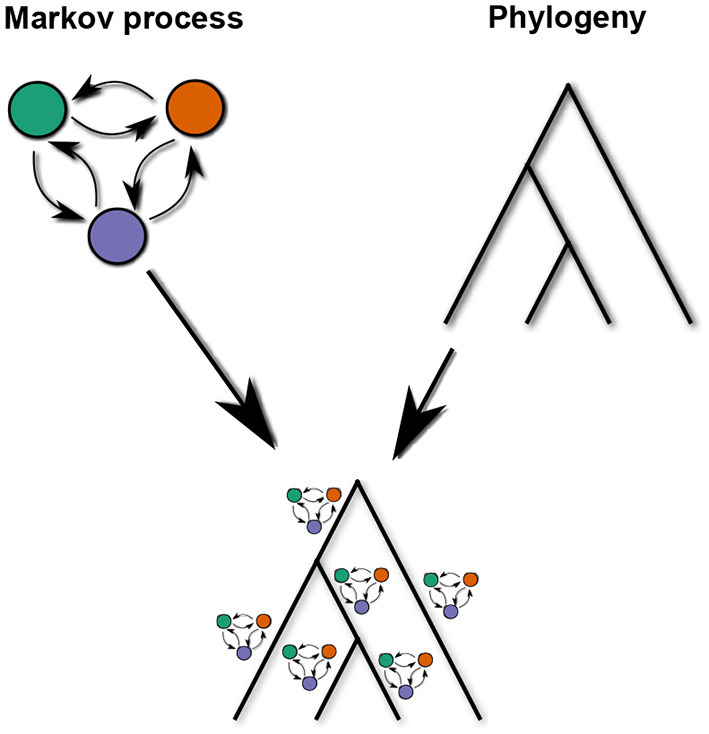
Schematic structure of the phylogenetic CTMC model. Independent but identical instances of a CTMC run on the branches of a phylogeny.

Let us illustrate this with an example. Suppose the feature in question has three possible values, *a*, *b*, and *c*. The *Q*-matrix characterizing the CTMC is given in (1).

(1) $$Q=\left(\begin{array}{rrr} -3 & 2 & 1\\ 5 & -6 & 1\\ 2 & 3 & -5 \end{array}\right)$$

The equilibrium distribution^5^ π for this CTMC is

(2) $$\begin{array}{l} \pi =(9/16,13/48,1/6)\\ \;\approx (0.56,0.27,0.17) \end{array}$$

The transition rates and the equilibrium distribution are illustrated in the upper panels of Figure 2.

**Figure 2:**
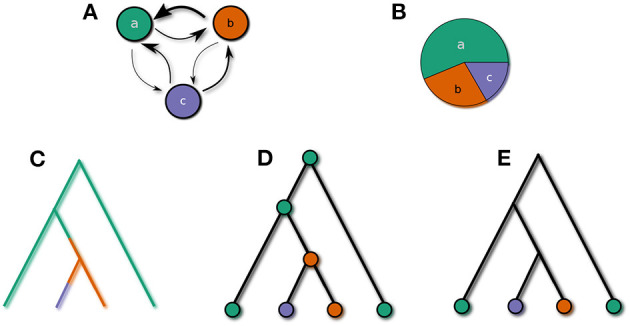
**(A)** CTMC, **(B)** equilibrium distribution, **(C)** fully specified history of a phylogenetic Markov chain, **(D)** Marginalizing over events at branches, **(E)** marginalizing over states at internal nodes.

A complete history of a run of this CTMC along the branches of a phylogeny is shown in Figure 2C. If the transition rates and branch lengths are known, the likelihood of this history, conditional on the state at the root, can be computed. To completely specify the likelihood of the history, one needs the probability distribution over states at the root of the tree—i.e., at the proto-language. In this paper we assume that proto-languages of known language families are the result of a long time of language change. If nothing about this history is known, the distribution of states at the proto-language is therefore virtually identical to the equilibrium distribution^6^.

The precise points in time where state transitions occur are usually unknown though. We can specify an infinite set of possible histories which only agree on the states of the nodes of the tree (illustrated in Figure 2D). The marginal likelihood of this set is the product of the conditional likelihood of the bottom node of each branch given the top node and the length of each branch, multiplied with the equilibrium probability of the root state.

Under normal circumstances only the states of the extant languages, i.e., at the leaves of the tree, are known. The marginal likelihood of all histories agreeing only in the states at the leaves can be determined by summing over all possible distribution of states at internal nodes (illustrated in Figure 2E). This quantity can be computed efficiently via a recursive bottom-up procedure known as Felsenstein's (1981) *pruning algorithm*.

This can easily be extended to sets of phylogenies (e.g., a collection of phylogenies for different language families; schematically illustrated in Figure 3). Language isolates are degenerate phylogenies with only one leave that is also the root. The likelihood of the state of an isolate is thus the equilibrium probability of its state.

**Figure 3:**
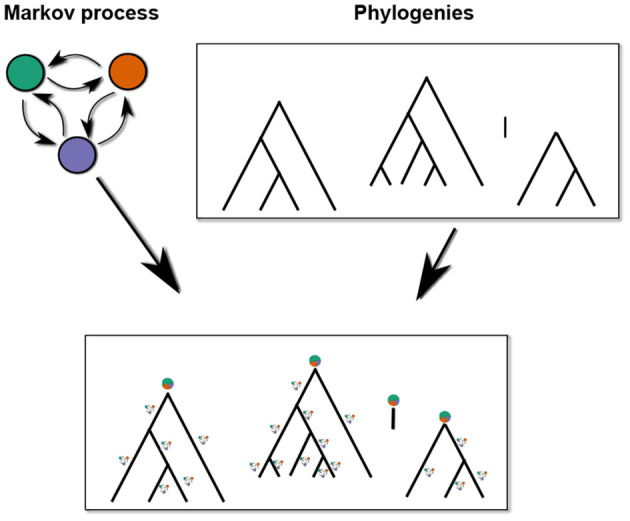
Phylogenetic Markov CTMC with a collection of phylogenies.

Under the assumption that the distributions in different language families are independent, the total likelihood of such a collection of phylogenies is the product of the individual tree likelihoods.

Under realistic conditions, the precise phylogeny of a language family is never known. Rather, it is possible to infer a probability distribution over phylogenies using Bayesian inference and, e.g., lexical data. In such a scenario, the *expected likelihood* for a language family is the averaged likelihood over this distribution of trees.

If only the phylogeny and the states at the leaves are known, statistical inference can be used to determine the transition rates (and thereby also the equilibrium distribution). Bayesian inference, that is used in this study, requires to specify prior assumptions over the transition rates and results in a posterior distribution over these rates.

In the remainder of this paper, this program is illustrated with a case on word order features and their potential correlations.

### 2.3. Word Order Features

The typical order of major syntactic constituents in declarative sentences of a language, and the distribution of these properties across the languages of the world, has occupied the attention of typologists continuously since the work of Greenberg (1963) (see, e.g., Lehmann, 1973; Vennemann, 1974; Hawkins, 1983; Dryer, 1992, among many others). There is a widespread consensus that certain word-order features are typologically correlated. For instance, languages with verb-object order tend to be prepositional while object-verb languages are predominantly postpositional. Other putative correlations, like the one between verb-object order and adjective-noun order, are more controversial.

The study in Dunn et al. (2011) undermined this entire research program. They considered eight word-order features and four major language families. For each pair of features and each family, they conducted a statistical test whether the feature pair is correlated in that family, using Bayesian phylogenetic inference. Surprisingly, they found virtually no agreement across language families. From this finding they conclude that the dynamics of change of word-order features is lineage specific; so the search for universals is void.

We will take up this problem and will consider the same eight word order features, which are taken from the *World Atlas of Language Structures* (WALS; Dryer and Haspelmath, 2013). For each of the 28 feature pairs, we will test two hypotheses:

1. All lineages (language families and isolates) share the parameters of a CTMC governing the evolution of these features (vs. Each lineage has its own CTMC parameters), and
2. If all lineages share CTMC parameters, the two features are correlated.

For each of the eight features considered, only the values “head-dependent” vs. “dependent-head” are considered. Languages that do not fall in either category are treated as “missing value”. These features and the corresponding values are listed in Table 1.

**Table 1:** Word order features.

**Feature**	**Value 1**	**Value 2**
VS	Verb-subject	Subject-verb
VO	Verb-object	Object-verb
PN	Adposition-noun	Noun-adposition
NG	Noun-genitive	Genitive-noun
NA	Noun-adjective	Adjective-noun
ND	Noun-demonstrative	Demonstrative-noun
NNum	Noun-numeral	Numeral-noun
NRc	Noun-relative clause	Relative clause-noun

### 2.4. Obtaining Language Phylogenies

Applying the phylogenetic Markov chain model to typological data requires phylogenies of the languages involved. In this section we describe how these phylogenies were obtained.

In Jäger (2018), a method is described how to extract binary characters out of the lexical data from the *Automated Similarity Judgment Program* (ASJP v. 18; Wichmann et al., 2018). These characters are suitable to use for Bayesian phylogenetic inference.

The processing pipeline described in Jäger (2018) is briefly recapitulated here. The ASJP data contains word lists from more than 7,000 languages and dialects, covering the translations of 40 core concepts. All entries are given in a uniform phonetic transcription.

In a first step, mutual string similarities are computed using pairwise sequence alignment along the lines of Jäger (2013). From these similarities, pairwise language distances are computed. These two measures are used to group the words for a each concept into cluster approximating *cognate classes*. Each such cluster defines a binary character, with value 1 for languages containing an element of the cluster in its word list, 0 if the entries for the same concepts all belong to different clusters, and undefined if there is no entry for that concept.

An additional class of binary characters is obtained from the Cartesian product of the 40 concepts and the 41 sound classes used in ASJP. A language has entry 1 for character “concept c/sound class s” if one of the entries for concept “c” contains at least one occurrence of sound class “s,” 0 if none of the entries for “c” contain “s,” and undefined if there is no entry for that concept.

In Jäger (2018), it is demonstrated that phylogenetic inference based on these characters is quite precise. For this assessment, the expert classification from Glottolog (Hammarström et al., 2018) is used as gold standard.

For the present study, we identified a total of 1,626 of languages for which WALS contains information about at least one word-order feature and the data from Jäger (2018) contain characters. These languages comprise 175 lineages according to the Glottolog classification, including 81 isolates^7^. The geographic distribution of this sample is shown in Figure 4.

**Figure 4:**
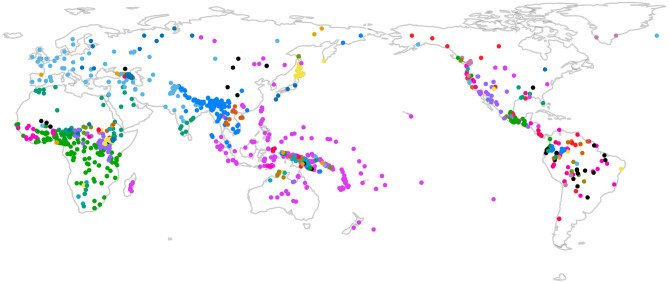
Geographic distribution of the sample of languages used. Colors indicate Glottolog classification.

Here, we used the cognate classes occurring within the language sample, as well as the concept/sound class characters as input for Bayesian phylogenetic inference. For each language family, a posterior tree sample was inferred using the Glottolog classification as constraint trees^8^. For each family, we sampled 1,000 phylogenies from the posterior distribution for further processing.

### 2.5. Generative Models

To study the co-evolution of two potentially correlated word-order features, we assume a four-state CTMC for each pair of such features—one state for each combination of values. We assume that all twelve state transitions are *a priori* possible, including simultaneous changes of both feature values^9^. The structure of the CTMC is schematically shown in Figure 5 for the feature pair VO/NA.

**Figure 5:**
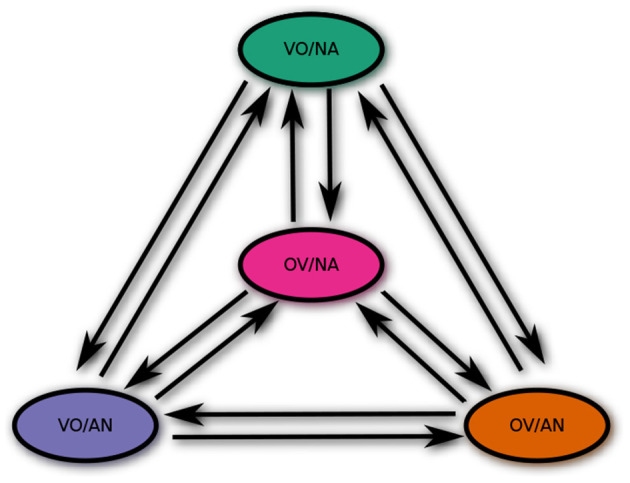
CTMC for a possibly correlated feature pair.

As pointed out above, Dunn et al. (2011) argue that the transition rates between the states of word-order features follow lineage-specific dynamics. To test this assumption (Hypothesis 1 above), we fitted two models for each feature pair:

- a **universal model** where all lineages follow the same CTMC with universally identical transition rates, and
- a **lineage-specific model** where each lineage has its own set of transition rate parameters.

These two model structures are illustrated in Figure 6.

**Figure 6:**
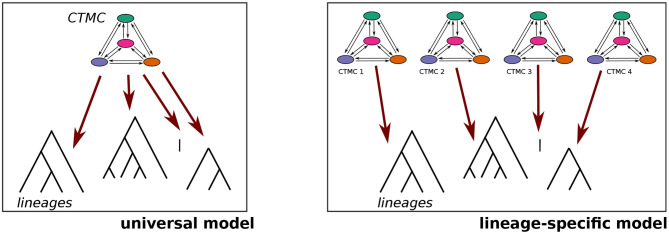
Universal vs. lineage-specific model.

For all models we chose a log-normal distribution with parameters μ = 0 and σ = 1 as prior for all rate parameters.

We will determine via statistical model comparison for each feature pair which of the two models fits the data better.

### 2.6. Prior Predictive Sampling

In a first step, we performed prior predictive sampling for both model types. This means that we simulated artificial datasets that were drawn from the prior distributions, and then compared them along several dimensions with the empirical data. This step is a useful heuristics to assess whether the chosen model and the chosen prior distributions are in principle capable to adequately model the data under investigation.

We identified three statistics to summarize the properties of these artificial data and compare them with the empirically observed data. For this purpose we represented each language as a probability vector over the four possible state. Let *Y* be the data matrix with languages as rows and states as columns, and *n* the number of languages, and *F* the set of lineages, where each lineage is a set of languages. The statistics used are:

- the **total variance**:
  $$\frac{1}{n}\sum_{i}\left(\sum_{l}Y_{l,i}^{2}-{\left(\sum_{l}Y_{l,i}\right)}^{2}\right)$$
- the **mean lineage-wise variance**:
  $$\frac{1}{\left|F\right|}\sum_{k}\frac{1}{\left|F_{k}\right|}\sum_{i}\left(\sum_{l\in F_{k}}Y_{l,i}^{2}-{\left(\sum_{l\in F_{k}}Y_{l,i}\right)}^{2}\right)$$
- the **cross-family variance**, i.e., the total variance between the centroids for each lineage:
  $$\sum_{i}\left(\frac{1}{\left|F\right|}\sum_{k}{\left(\frac{1}{\left|F_{k}\right|}\sum_{l\in F_{k}}Y_{l,i}\right)}^{2}-{\left(\frac{1}{\left|F\right|}\sum_{k}\frac{1}{\left|F_{k}\right|}\sum_{l\in F_{k}}Y_{l,i}\right)}^{2}\right)$$

In Figure 7, the distribution of these statistics for the 28 feature pairs for the empirical data are compared with the prior distributions for the universal model (top panels) and the lineage-specific model (bottom panels). For each model, we conducted 1,000 simulation runs.

**Figure 7:**
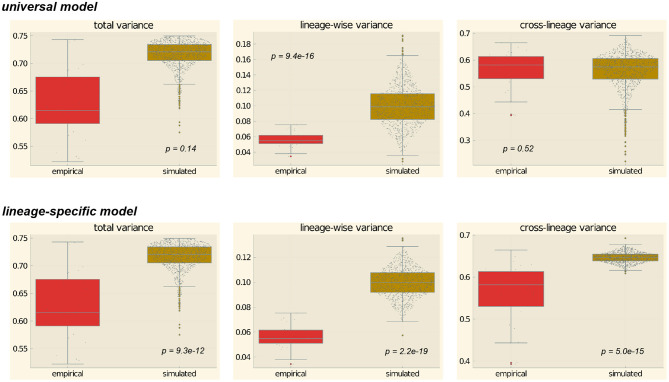
Prior predictive simulations. The *p*-values are the result of a Mann-Whitney *U*-test whether empirical and simulated values come from the same distribution.

From visual inspection it is easy to see that for the universal model, the empirically observed values fall squarely within the range of the prior distributions. For the lineage-specific model, the observed variances are generally lower than expected under the prior distribution. This is especially obvious with regard to the cross-family variance, which is much lower for the empirical data than predicted by the model.

Following the suggestion of a reviewer, we performed a Mann-Whitney *U*-test for each configuration to test the hypothesis that empirical and simulated data come from the same distribution. The results (shown inside the plots in Figure 7) confirm the visual inspection. For the total variance and the cross-lineage variance and the universal model, the hypothesis of equal distributions cannot be rejected, while the empirical distribution differs significantly from the simulated data for the other four configurations.

### 2.7. Model Fitting

Both models were fitted for each of the 28 feature pairs. Computations were performed using the programming language *Julia* and Brian J. Smith's package *Mamba* (https://github.com/brian-j-smith/Mamba.jl) for Bayesian inference. We extended *Mamba* by functionality to handle phylogenetic CTMC models.

Posterior samples were obtained via Slice sampling (Neal, 2003). Averaging over the prior of phylogenies was achieved by randomly picking one phylogeny from the prior (see section 2.4) in each MCMC step. Posterior sampling was stopped when the *potential scale reduction factor* (PSRF; Gelman and Rubin, 1992) was ≤ 1.1 for all parameters.

### 2.8. Posterior Predictive Sampling

To test the fit of the models to the data, we performed *posterior predictive sampling* for all fitted models. This means that for each model, we randomly picked 1,000 samples from the posterior distribution and used it to simulate artificial datasets. The three statistics used above for prior predictive sampling were computed for each simulation. The results are shown in the Supplementary Material.

With regard to total variance, we find that the empirical value falls outside the 95% highest posterior interval for three out of 28 feature pairs (VO-NRc, PN-NRc, and NA-ND), where the model overestimates the total variance. The lineage-specific model overestimates the total variance for 10 feature pairs.

Since three outliers out of 28 is within the expected range for a 95% interval, we can conclude that the universal model generally predicts the right amount of cross-linguistic variance. The lineage-specific model overestimates this quantity.

For cross-linguistic variance, the empirical value falls outside the HPD (95% highest posterior density interval) for 14 pairs for the universal model and for 21 pairs for the lineage-specific model. So both models tend to overestimate this variable. This might be due to the fact that phylogenetic CTMC models disregard the effect of language contact, which arguably reduced within-family variance.

The cross-family variance falls into the universal model's HPD for all pairs, but only for two pairs (VO-NA, VO-NNum) for the lineage-specific model. Briefly put, the universal model gets this quantity right while the lineage-specific model massively overestimates it.

### 2.9. Bayesian Model Comparison

As a next step we performed statistical model comparison between the universal and the lineage-specific model. Briefly put, model comparison estimates how well models will serve to predict unseen data that are generated by the same process as the observed data, and compares the predictive performances. Everything else being equal, the model with the better predictive performance can be considered a better explanation for the observed data.

Since there is no general consensus about the best method to compare Bayesian models (see, e.g., Vehtari and Ojanen, 2012 for an overview), we applied two techniques.

The *marginal likelihood* of the data under a Bayesian model is the expected likelihood of the data *y* weighted by the prior probability of the model parameters θ.

$$D(y|M)=\int_{\theta }p(y|\theta )p(\theta |M)d\theta$$

The Bayes factor between two models *M*_1_ and *M*_2_ is the ratio of their marginal densities:

$$BF=\frac{D(y|M_{1})}{D(y|M_{2})}$$

To estimate the marginal densities, we used **bridge sampling** (cf. Gronau et al., 2017). For our implementation we depended strongly on the *R*-package *bridgesampling* (Gronau et al., 2020). The logarithmically transformed Bayes factors between the universal model (≈ *M*_1_) and the lineage-specific model (≈ *M*_2_) are shown for each feature pair in Table 2.

**Table 2:** log-Bayes factor between universal and lineage-specific model.

**Feature pair**	**(log) Bayes factor**	**Cumulative posterior probability**
VS-VO	72.9	0.000
VS-NG	65.9	0.000
PN-NG	64.5	0.000
VO-PN	56.8	0.000
VS-PN	54.4	0.000
VO-NG	41.3	0.000
VS-NRc	36.5	1.11e-16
NA-ND	32.1	1.18e-14
VS-NNum	31.1	4.19e-14
VS-NA	30.8	8.57e-14
NG-NRc	28.0	8.09e-13
VO-NRc	27.7	1.79e-12
VS-ND	27.0	3.67e-12
PN-NRc	25.6	1.12e-11
NA-NRc	22.1	2.63e-10
NG-ND	19.0	5.98e-9
NG-NA	18.8	1.29e-8
ND-NNum	15.6	1.76e-7
PN-NA	15.2	4.38e-7
NA-NNum	8.8	0.000147
PN-ND	8.7	0.000319
ND-NRc	7.3	0.00101
NG-NNum	6.7	0.00223
VO-ND	6.4	0.00393
NNum-NRc	5.3	0.00892
PN-NNum	5.1	0.0152
VO-NA	5.0	0.0218
VO-NNum	2.4	0.102

*The last row gives the upper limit of the posterior probability that for at least one feature-pair up to this line the lineage-specific model is true*.

All log-Bayes factors are positive, i.e., favor the universal over the lineage-specific model.

According to the widely used criteria by Jeffreys (1998), a Bayes factor of ≥ 100, which corresponds to a logarithmic Bayes factor of 4.6, is considered as decisive evidence. So except for the feature pair VO-NNum, this test provides decisive evidence in favor of the universal model.

Unlike frequentist hypothesis testing, Bayesian model comparison does not require a correction for multiple testing. Still, since 28 different hypotheses are tested simultaneously here, the question arises how confident we can be that a given subset of the hypotheses are true. Assuming the uninformative prior that the universal and the lineage-specific model are equally likely *a priori*, the posterior probability of the universal model being true given that one of the two models is true, is the logistic transformation of the log-Bayes factor. Let us call this quantity $p_{i}^{u}$ for feature pair *i*. We assume that feature-pairs are sorted in descending order according to their Bayes factor, as in Table 2. The posterior probability of the lineage-specific model is $p_{i}^{l}=1-p_{i}^{u}$. The quantity $p_{1\cdots k}^{l}$ is the cumulative probability that the lineage-specific model is true for at least one feature pair *i* with 1 ≤ *i* ≤ *k*^10^.

Since the hypotheses for the individual feature pairs are not mutually independent, it is not possible to compute this probability. However, according to the *Bonferroni inequality*, it holds that

$$p_{1\cdots k}^{l}\leq \sum_{1\leq i\leq k}p_{k}^{l}$$

The right-hand side of this inequality provides an upper limit for the left-hand side. This upper limit is shown in the third column of Table 2. For all but the feature pair VO-NNum, this probability is < 0.05. We conclude that this line of reasoning also confirms that the data strongly support the universal over the lineage-specific hypothesis for all feature pairs except VO-NNum.

As alternative approach to model comparison, we conducted **Pareto-smoothed cross-validation** (Vehtari et al., 2017) using the *R*-package *loo* (Vehtari et al., 2020).

Leave-one-out cross-validation means to loop through all data points *y*_*i*_ and compute the quantity

$$\log p(y_{i}|y_{-i})=\int_{\theta }p(y_{i}|\theta )p(\theta |y_{-i})d\theta$$

Here, *y*_−*i*_ denotes the collection of all datapoints ≠ *y*_*i*_. Since this amounts to fitting a posterior distribution as often as there are datapoints, this is computationally not feasible in most cases (including the present case study). The quantity

$$\text{elpd}=\sum_{i}\log p(y_{i}|y_{-i}),$$

the *expected log pointwise predictive density*, is a good measure of how well a model predicts unseen data and can be used to compare models.

Since computing the elpd amounts to fitting a posterior distribution for each datapoint, the method is not feasible though in most cases (including the present case study). Pareto-smoothed leave-one-out cross-validation is a technique to estimate elpd from the posterior distribution of the entire dataset.

However, this algorithm depends on the assumption that individual datapoints are mutually *conditionally independent*, i.e.,

$$p(y|\theta )=\prod_{i}p(y_{i}|\theta ).$$

This is evidently not the case for phylogenetic CTMC models if we treat each language as a datapoint^11^. However, conditional independence does hold between lineages both in the universal and the lineage-dependent model. Pareto-smoothed leave-one-out cross-validation can be therefore be performed if entire lineages are treated as datapoints.

The difference in elpd, i.e., elpd of universal model minus elpd of lineage-specific model, are shown in Table 3. For all feature pairs, the elpd is higher for the universal than for the lineage-specific model.

**Table 3:** Differences in elpd.

**Feature pair**	**Δ elpd**
VS-VO	79.7
PN-NG	75.9
VS-NG	72.6
VO-PN	65.3
VS-PN	61.4
VO-NG	48.3
VS-NRc	45.5
NA-ND	37.3
NG-NRc	36.7
VS-NA	35.3
VO-NRc	34.6
PN-NRc	32.7
VS-NNum	28.7
NA-NRc	27.9
VS-ND	26.1
NG-NA	21.1
PN-ND	19.2
PN-NA	18.0
NG-ND	16.1
VO-ND	13.6
ND-NNum	12.5
NG-NNum	12.3
ND-NRc	7.5
PN-NNum	6.6
NA-NNum	4.2
NNum-NRc	3.7
VO-NNum	3.5
VO-NA	1.1

To summarize, for all feature pairs except VO-NNum, different methods of model comparison agree that the universal model provides a better fit to the data than the lineage-specific model. For VO-NNum, the evidence is more equivocal, but it is also slightly favors the universal model.

From this we conclude that there is no sufficient empirical evidence for the assumption of lineage specificity in the evolution of correlated word-order features. Dunn et al.'s (2011) finding to the contrary is based on a much smaller sample of 301 languages from just four families, and it omits an explicit model comparison.

### 2.10. Feature Correlations

Let us know turn to the second hypothesis mentioned in section 2.3, repeated here. For each feature pair, we will probe the question:

If all lineages share CTMC parameters, the two features are correlated.

To operationalize correlation, we define the feature value “dependent precedes head” as 0 and “head precedes dependent” as 1. For a given feature pair, this defines a 2 × 2 contingency table with posterior equilibrium probabilities for each value combination. They are displayed in Figure 8. In each diagram, the *x*-axis represents the first feature and the *y*-axis the second feature. The size of the circles at the corners of the unit square indicate the equilibrium probability of the corresponding value combination. Blurred edges of the circles represent posterior uncertainty.

**Figure 8:**
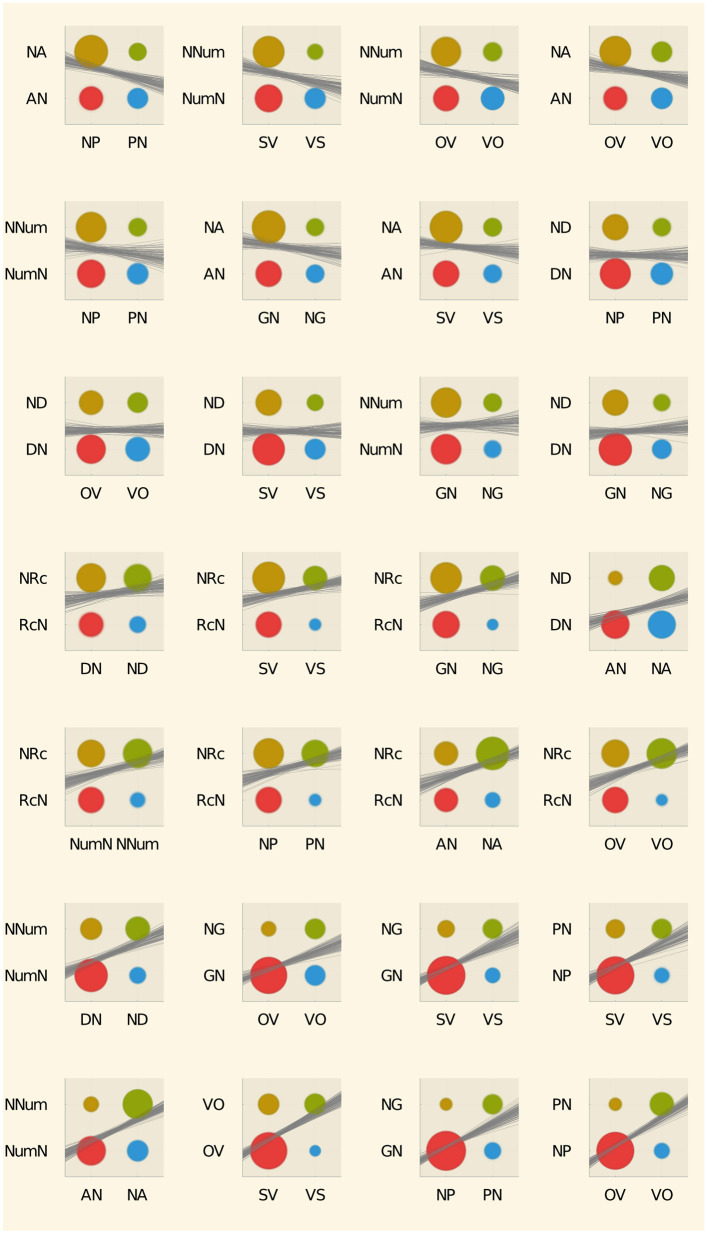
Posterior equilibrium probabilities and linear regression.

The diagrams also show the posterior distribution of regression lines indicating the direction and strength of the association between the two features^12^. Perhaps surprisingly, for some feature pairs the association is negative.

The *correlation* between two features binary *f*_1_, *f*_2_ in the strict mathematical sense, also called the *Phi coefficient*, is

$$\begin{array}{l} \;\frac{\text{cov}(f_{1},f_{2})}{\sqrt{\text{var}(f_{1})\text{var}(f_{2})}}\\ =\frac{p_{00}p_{11}-p_{10}p_{01}}{\sqrt{\left(p_{00}+p_{01})(p_{10}+p_{11})(p_{00}+p_{10})(p_{10}+p_{11}\right)}} \end{array}$$

and ranges from −1 (perfect negative relationship) to 1 (perfect positive relationship), with 0 indicating no relationship.

The median posterior correlations and the corresponding HPD interval given in Table 4 and shown in Figure 9.

**Table 4:** Correlation coefficients for feature pairs: median and 95% HPD interval.

**Feature pair**	**Median**	**HPD**
VO-PN	0.64	(0.53, 0.75)
PN-NG	0.55	(0.41, 0.67)
VS-VO	0.49	(0.38, 0.60)
NA-NNum	0.47	(0.34, 0.59)
VS-PN	0.45	(0.32, 0.58)
VS-NG	0.45	(0.32, 0.58)
VO-NG	0.41	(0.27, 0.53)
ND-NNum	0.38	(0.26, 0.50)
VO-NRc	0.38	(0.24, 0.50)
NA-NRc	0.37	(0.23, 0.51)
PN-NRc	0.28	(0.14, 0.42)
NNum-NRc	0.28	(0.13, 0.42)
NA-ND	0.27	(0.15, 0.39)
NG-NRc	0.24	(0.09, 0.38)
VS-NRc	0.19	(0.05, 0.32)
ND-NRc	0.17	(0.00, 0.32)
NG-ND	0.06	(−0.06, 0.20)
NG-NNum	0.05	(−0.09, 0.19)
VS-ND	−0.00	(−0.13, 0.14)
VO-ND	−0.01	(−0.13, 0.12)
PN-ND	−0.01	(−0.15, 0.11)
VS-NA	−0.09	(−0.22, 0.05)
NG-NA	−0.12	(−0.24, 0.02)
PN-NNum	−0.12	(−0.27, 0.05)
VO-NA	−0.17	(−0.30, −0.04)
VO-NNum	−0.19	(−0.32, −0.05)
VS-NNum	−0.20	(−0.33, −0.06)
PN-NA	−0.21	(−0.34, −0.08)

**Figure 9:**
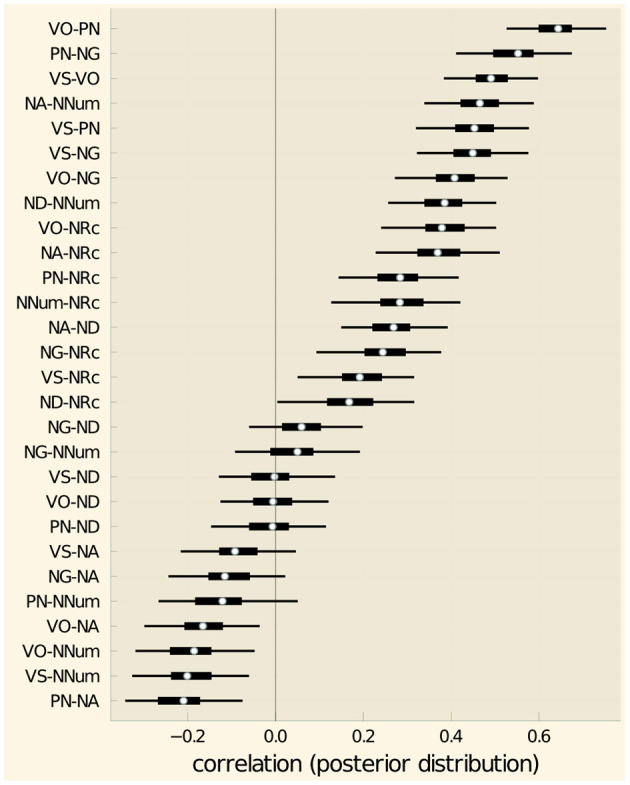
Correlation coefficients for feature pairs. White dots indicate the median, thick lines the 50% and thin lines the 95% HPD intervals.

How reliable are these estimates? The Bayes factor between the hypotheses “correlation ≠ 0” and “correlation = 0” can be determined via the Savage-Dickey method (Dickey and Lientz, 1970; see also Wagenmakers et al., 2010). We used the *R*-package *LRO.utilities* (https://github.com/LudvigOlsen/LRO.utilities/) to carry out the computations. The log-Bayes factors for the individual feature pairs are shown in Table 5.

**Table 5:** log-Bayes factor between “correlation ≠ 0” and “correlation = 0”.

**Feature pair**	**(log) Bayes factor**	**Cumulative posterior probability**
VO-PN	19.25	**4.37e-9**
VS-VO	15.82	**1.39e-7**
VS-NG	14.12	**8.78e-7**
PN-NG	12.07	**6.62e-6**
VS-PN	12.03	**1.26e-5**
NA-NNum	10.93	**3.05e-5**
ND-NNum	9.98	**7.68e-5**
VO-NG	8.85	**0.00022**
VO-NRc	8.15	**0.000509**
NA-NRc	7.43	**0.0011**
NA-ND	4.72	**0.00995**
NNum-NRc	4.07	**0.0267**
PN-NRc	3.83	**0.0479**
PN-NA	3.17	0.0884
NG-NRc	2.64	0.155
VS-NNum	2.47	0.233
VO-NNum	2.01	0.351
VS-NRc	1.93	0.478
VO-NA	1.60	0.646
ND-NRc	0.55	1.000
NG-NA	−0.03	1.000
PN-NNum	−0.43	1.000
VS-NA	−0.51	1.000
NG-ND	−1.17	1.000
NG-NNum	−1.32	1.000
PN-ND	−1.52	1.000
VO-ND	−1.56	1.000
VS-ND	−1.64	1.000

*The last row gives the upper limit of the posterior probability that for at least one feature-pair up to this line correlation = 0. Cumulative posterior probabilites < 0.05 are shown in bold*.

Using the same method as in section 2.9, we can conclude with 95% confidence that there is a non-zero correlation for 13 feature pairs: VO-PN, VS-VO, VS-NG, PN-NG, NA-NNum, ND-NNum, VO-NG, VO-NRc, NA-NRc, NA-ND, NNum-NRc, PN-NRc. For all these pairs, the correlation coefficient is credibly positive (meaning the 95% HPD interval is entirely positive). There is not sufficient evidence that there is a negative correlation for any feature pair. For the four feature pairs where the HPD interval for the correlation coefficient is entirely negative (VO-NA, VO-NNum, VS-NNum, PN-NA), the log-Bayes factors in favor of a non-zero correlation (1.60, 2.01, 2.47, 3.17) are too small to merit a definite conclusion.

Conversely, for no feature pair is the Bayes factor in favor of a zero-correlation large enough to infer the absence of a correlation.

## 3. Discussion

### 3.1. Equilibrium Analysis vs. Language Sampling

Maslova (2000) argues that the frequency distribution of typological feature values may be biased by accidents of history, and that the equilibrium distribution of the underlying Markov process more accurately reflects the effects of the cognitive and functional forces. Inspection of our results reveals that the difference between raw frequencies and equilibrium probabilities can be quite substantial. In Table 6, the relative frequencies, the equilibrium frequencies and the 95% HPD intervals for the four values of the feature combination “verb-object/adposition-noun” are shown.

**Table 6:** Relative frequencies, stratified frequencies, and posterior probabilities of the four value combinations of VO-PN.

**Values**	**Relative frequencies**	**Stratified relative frequencies**	**Equilibrium (median)**	**HPD**
OV-NAdp	0.420	0.663	0.614	(0.540, 0.685)
OV-AdpN	0.011	0.009	0.060	(0.032, 0.096)
VO-NAdp	0.030	0.051	0.091	(0.054, 0.134)
VO-AdpN	0.540	0.278	0.230	(0.175, 0.293)

We also computed the *stratified frequencies*, i.e., the weighted means where each language is weighted by the inverse of the size of its Glottolog lineage. As a result, each lineage has the same cumulative weight.

The same information is displayed in Figure 10. It can be discerned that uniformly head-initial languages (VO-AdpN) are over-represented among the languages of the world in comparison to the equilibrium distribution while uniformly head-final languages (OV-NAdp) are underrepresented. The stratified frequencies come very close to the equilibrium distribution though. This discrepancies are arguably due to the fact that head-initial languages are predominant in several large families while head-final languages are quite frequent among small families and isolates.

**Figure 10:**
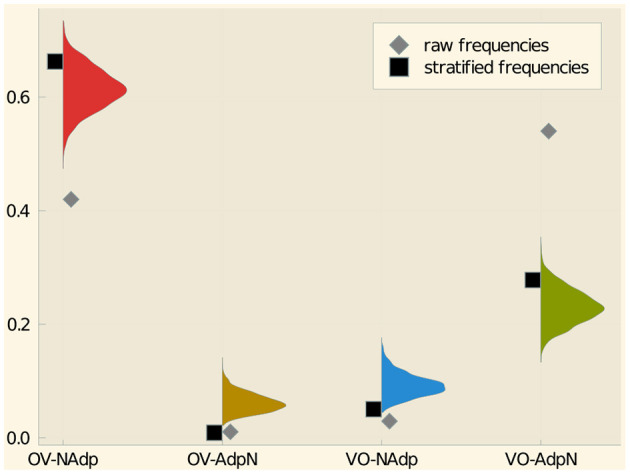
Relative frequencies, stratified frequencies, and posterior probabilities of the four value combinations of VO-PN.

This example suggests that our approach effectively achieves something similar than stratified sampling, namely discounting the impact of large families and give more weight to small families and isolates. A more detailed study of the relationship between stratified sampling and equilibrium analysis is a topic for future research.

### 3.2. Universal vs. Lineage-Specific Models

The findings from section 2.9 clearly demonstrate that the universal model provides a better fit of the data than the lineage-specific model. This raises the question why Dunn et al. (2011) came to the opposite conclusion. There are several relevant considerations. First, these authors did not directly test a universal model. Rather, they fitted two lineage-specific models for each feature pair—one where the features evolve independently and one where the mutation rates of one feature may depend on the state of the other feature. They then compute the Bayes factor between these models for each family separately and conclude that the patterns of Bayes factors vary wildly between families. So essentially it is tested whether the pattern of feature correlations is identical across families.

In this paper, we explored slightly different hypotheses. We tested whether the data support a model where all lineages following the same dynamics with the same parameters (where a correlation between features is possible), or whether they support different parameters (each admitting a correlation between features). Having the same model across lineages implies an identical correlation structure, but it also implies many other things, such as identical equilibrium distributions, identical rate of change etc.

To pick an example, Dunn et al. (2011) found evidence for a correlation between NA and NRc for Austronesian and Indo-European but not for Bantu and Uto-Aztecan. This seems to speak against a universal model. However, inspection of our data reveals that the feature value “relative clause precedes noun” only occurs in 1.8% of all Austronesian and 13.8% of all Indo-European languages, and it does not occur at all in Bantu or Uto-Aztecan. The universal model correctly predicts that the observed frequency distributions will be similar across lineages (as demonstrated by the low cross-family variance in the prior predictive simulations discussed in section 2.6). The lineage-specific model cannot account for this kind of cross-family similarities. More generally, our approach to test the relative merits of a universal versus lineage-specific dynamics regarding word-order features takes more sources of information into account than just correlation patterns. This more inclusive view clearly supports the universal model.

### 3.3. Word-Order Correlations

The 13 feature pairs identified in section 2.10 for which there is credible evidence for a correlation are shown in Figure 11, where connecting lines indicate credible evidence for a correlation.

**Figure 11:**
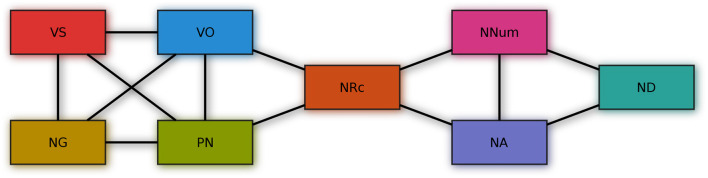
Feature-pairs with credible evidence for a correlation.

The four features correlated with VO are exactly those among the features considered here that were identified by Dryer (1992) as “verb patterners,” i.e., for which he found evidence for a correlation with verb-object order. These are verb-subject, noun-genitive, adposition-noun and noun-relative clause. It is perhaps noteworthy that like Dryer, we did not find credible evidence for a correlation between verb-object order and noun-adjective order, even though such a connection has repeatedly been hypothesized, e.g., by Lehmann (1973) and Vennemann (1974), and, more recently, by Ferrer-i-Cancho and Liu (2014).

Besides Dryer's verb patterns, we found a group of three mutually correlated features, noun-numeral, noun-adjective and noun-demonstrative. Two of them, noun-numeral and noun-adjective, are also correlated with noun-relative clause. These correlations have received less attention in the typological literature. The findings are not very surprising though, given that all these features pertain to the ordering of noun-phrase material relative to the head noun.

## 4. Conclusion

In this article we demonstrated that the modeling of typological feature distributions in terms of phylogenetic continuous-time Markov chains—inspired Maslova's (2000) theoretical work as well as by research within the framework of the biological comparative method such as Pagel and Meade (2006) and Dunn et al. (2011)—has several advantages for typology. It allows to use all data, from families large and small as well as from isolate languages. The method controls for non-independence due to common descent. Couched in a Bayesian framework, it affords standard techniques for model checking and model comparison as well as quantification of the uncertainty in inference. We do see it as essential though that this kind of study uses data from a variety of lineages since individual families generally do not display evidence for all the possible diachronic transitions required to estimate transition rates reliably. Working with forests rather than single trees, i.e., with trees or tree distributions for several families and also including isolates as elementary trees is a suitable way to achieve this goal^13^.

To demonstrate the viability of this method, we chose a re-assessment of the issue broad up by Dunn et al. (2011): Are word-order correlations lineage specific or universal? Using a collection of 1,626 languages from 175 lineages (94 families and 81 isolates), we found conclusive evidence that a universal model provides a much better fit to the word-order data from WALS than a lineage-specific model. Furthermore we found statistical evidence for a correlation for 13 word-order features (out of 28 considered), which largely confirm the findings of traditional typological research.

There is a variety of open issues for future research. Maslova (2000) also discusses the possibility that the current distribution of feature value represents traces of proto-world or some later bottleneck language, which would bias the estimation of the equilibrium distribution. In the present paper this option was disregarded. It is possible to address this question using Bayesian model comparison.

By design, phylogenetic models only capture vertical transmission. The effects of language contact and areal tendencies are systematically ignored. In future work, this could be remedied by including areal and spatial random effects into the model.

Statistical research in other disciplines involving stratified data suggest that the binary alternative between a lineage-specific and a universal model might be ill-posed. Both approaches can be integrated within *hierarchical models* (see, e.g., Gelman et al., 2014; McElreath, 2016) where between-group variance is as small as possible but as large as the data require. Due to the high number of parameters involved, fitting such models, however, poses a considerable computational challenge.

## Data Availability Statement

Publicly available datasets were analyzed in this study. This data can be found at: http://asjp.clld.org/ (Automated Similarity Judgment Program); http://glottolog.org (Glottolog); http://wals.info/ (World Atlas of Language Structure); https://doi.org/10.17605/OSF.IO/CUFV7 (accompanying data for Jäger, 2018, Global-scale phylogenetic linguistic inference from lexical resources, Scientific Data). The code used for this study can be found at https://github.com/gerhardJaeger/phylogeneticTypology.

## Author Contributions

GJ conducted the study and wrote up the article. JW programmed the software used and gave technical support. All authors contributed to the article and approved the submitted version.

## Conflict of Interest

The authors declare that the research was conducted in the absence of any commercial or financial relationships that could be construed as a potential conflict of interest.

## Footnotes

^1^For example, Greenberg's Universal 1 “In declarative sentences with nominal subject and object, the dominant order is almost always one in which the subject precedes the object”.

^2^For instance Universal 3 “Languages with dominant VSO order are always prepositional”.

^3^In the context of the present study, this is taken to be a possibly simplifying assumption that is part of the statistical approach taken. Whether or not it is empirically true would require a more careful discussion than what is possible here. Our currently best guess is that the assumption holds true provided a sufficiently fine-grained notion of “state” is adopted. For the coarse-grained states taken from WALS, such as VO or AN, the assumption is arguably too simplistic.

^4^See, e.g., Grimmett and Stirzaker (2001).

^5^This is the left null vector of *Q*, normalized such that it sums to 1.

^6^It is possible to test for a given feature distribution and phylogeny whether the data support this hypothesis. We leave this issue for future work.

^7^A language is called an isolate here if our 1,626-language sample contains no other language belonging to the same Glottolog family.

^8^For this purpose we used the software *MrBayes* (Ronquist and Huelsenbeck, 2003) for families with at least three members and *RevBayes* (Höhna et al., 2016) for two-language families.

^9^Dunn et al. (2011), following the general methodology of Pagel and Meade (2006), exclude this possibility. We believe, however, that this possibility should not be excluded *a priori* because it is conceivable that during a major reorganization of the grammar of a language, several features change their values at once.

^10^This amounts to the Holm-Bonferroni correction (Holm, 1979), but we use it here to compute an upper limit for the posterior probability rather than for the expected α level.

^11^To see why, consider an extreme case where the phylogeny consists of two leaves with an infinitesimally small co-phenetic distance, and the equilibrium distribution over states is the uniform distribution. Then *p*(*y*_1_ = *y*_2_ = *s*_1_) ≈ *p*(*y*_1_ = *s*_1_) = *p*(*y*_2_ = *s*_1_) < *p*(*y*_1_ = *s*_1_)*p*(*y*_2_ = *s*_1_).

^12^Intercept and slope of the regression lines are $\frac{p_{01}}{p_{00}+p_{01}}$ and $\frac{p_{11}}{p_{10}+p_{11}}-\frac{p_{01}}{p_{00}+p_{01}}$, respectively.

^13^Verkerk et al. (2021) use a similar approach but utilize a universal tree encompassing all lineages.
