# Supplementary material for: Phylogenetic Typology
Source: Front Psychol. 2021 Jul 19;12:682132. doi: 10.3389/fpsyg.2021.682132 (PMC8326798; doi:10.3389/fpsyg.2021.682132)
Supplement: Supplementary file 1 [file Data_Sheet_1.pdf]

Supplementary Material

1 POSTERIOR PREDICTIVE SAMPLING

This section contains the tables and figures reporting the results of the posterior predictive sampling (Subsection 2.8 of the main text).

feature pair	empirical	universal model (HPD)	lineage-specific model (HPD)
VS-VO	0.614	**(0.410, 0.710)**	(0.669, 0.749)
VS-PN	0.621	**(0.337, 0.734)**	(0.661, 0.748)
VS-NG	0.598	**(0.339, 0.685)**	(0.672, 0.749)
VS-NA	0.561	**(0.560, 0.744)**	(0.672, 0.750)
VS-ND	0.609	**(0.558, 0.745)**	(0.669, 0.749)
VS-NNum	0.598	**(0.578, 0.748)**	(0.675, 0.749)
VS-NRc	0.576	**(0.573, 0.735)**	(0.662, 0.748)
VO-PN	0.531	**(0.358, 0.699)**	(0.660, 0.748)
VO-NG	0.611	**(0.413, 0.717)**	(0.671, 0.748)
VO-NA	0.672	**(0.564, 0.749)**	**(0.667, 0.750)**
VO-ND	0.688	**(0.634, 0.749)**	**(0.669, 0.750)**
VO-NNum	0.738	**(0.598, 0.748)**	**(0.671, 0.748)**
VO-NRc	0.538	(0.590, 0.747)	(0.656, 0.748)
PN-NG	0.569	**(0.270, 0.651)**	(0.665, 0.749)
PN-NA	0.671	**(0.513, 0.747)**	**(0.656, 0.750)**
PN-ND	0.691	**(0.582, 0.748)**	**(0.651, 0.749)**
PN-NNum	0.742	**(0.572, 0.747)**	**(0.663, 0.749)**
PN-NRc	0.528	(0.577, 0.739)	(0.634, 0.748)
NG-NA	0.686	**(0.518, 0.746)**	**(0.675, 0.749)**
NG-ND	0.698	**(0.537, 0.740)**	**(0.673, 0.748)**
NG-NNum	0.743	**(0.566, 0.747)**	**(0.674, 0.750)**
NG-NRc	0.596	**(0.566, 0.733)**	(0.661, 0.749)
NA-ND	0.612	(0.619, 0.747)	(0.664, 0.749)
NA-NNum	0.627	**(0.580, 0.743)**	(0.662, 0.749)
NA-NRc	0.522	**(0.518, 0.738)**	(0.647, 0.749)
ND-NNum	0.659	**(0.532, 0.748)**	(0.666, 0.750)
ND-NRc	0.616	**(0.606, 0.747)**	(0.646, 0.749)
NNum-NRc	0.664	**(0.611, 0.750)**	**(0.645, 0.749)**

Table S1. Posterior predictive simulation: Total variance. Intervals are shown in bold if they include the empirical value.

2 TRANSITION RATES: POSTERIOR DISTRIBUTIONS

A visualization of the magnitude of the transition rates for all feature pairs is shown in Figure S4.

The medians and HPD intervals can be seen in `https://github.com/gerhardJaeger/phylogeneticTypology/blob/main/data/posteriorStatistics.csv`.

feature pair	empirical	universal model (HPD)	lineage-specific model (HPD)
VS-VO	0.051	(0.066, 0.146)	(0.064, 0.106)
VS-PN	0.048	(0.051, 0.122)	(0.063, 0.108)
VS-NG	0.056	**(0.051, 0.121)**	(0.062, 0.104)
VS-NA	0.061	**(0.060, 0.149)**	(0.063, 0.105)
VS-ND	0.071	**(0.064, 0.140)**	**(0.060, 0.102)**
VS-NNum	0.056	(0.068, 0.163)	(0.062, 0.106)
VS-NRc	0.062	(0.077, 0.183)	**(0.055, 0.104)**
VO-PN	0.038	(0.047, 0.120)	(0.064, 0.111)
VO-NG	0.047	(0.050, 0.118)	(0.063, 0.108)
VO-NA	0.055	**(0.051, 0.127)**	(0.066, 0.110)
VO-ND	0.075	**(0.066, 0.146)**	**(0.065, 0.107)**
VO-NNum	0.054	**(0.051, 0.128)**	(0.065, 0.107)
VO-NRc	0.050	(0.063, 0.163)	(0.059, 0.107)
PN-NG	0.034	(0.038, 0.117)	(0.063, 0.111)
PN-NA	0.056	**(0.055, 0.131)**	(0.065, 0.110)
PN-ND	0.070	**(0.057, 0.135)**	(0.060, 0.107)
PN-NNum	0.054	**(0.049, 0.141)**	(0.064, 0.109)
PN-NRc	0.056	(0.058, 0.152)	(0.057, 0.107)
NG-NA	0.062	**(0.046, 0.126)**	(0.063, 0.108)
NG-ND	0.075	**(0.053, 0.127)**	**(0.061, 0.107)**
NG-NNum	0.054	**(0.043, 0.121)**	(0.063, 0.107)
NG-NRc	0.046	(0.060, 0.155)	(0.056, 0.108)
NA-ND	0.068	(0.072, 0.164)	**(0.064, 0.105)**
NA-NNum	0.049	**(0.049, 0.135)**	(0.063, 0.107)
NA-NRc	0.052	(0.054, 0.138)	(0.056, 0.105)
ND-NNum	0.070	**(0.046, 0.119)**	**(0.061, 0.106)**
ND-NRc	0.052	(0.058, 0.149)	(0.055, 0.103)
NNum-NRc	0.051	(0.058, 0.162)	(0.054, 0.107)

Table S2. Posterior predictive simulation: Lineage-wise variance. Intervals are shown in bold if they include the empirical value.

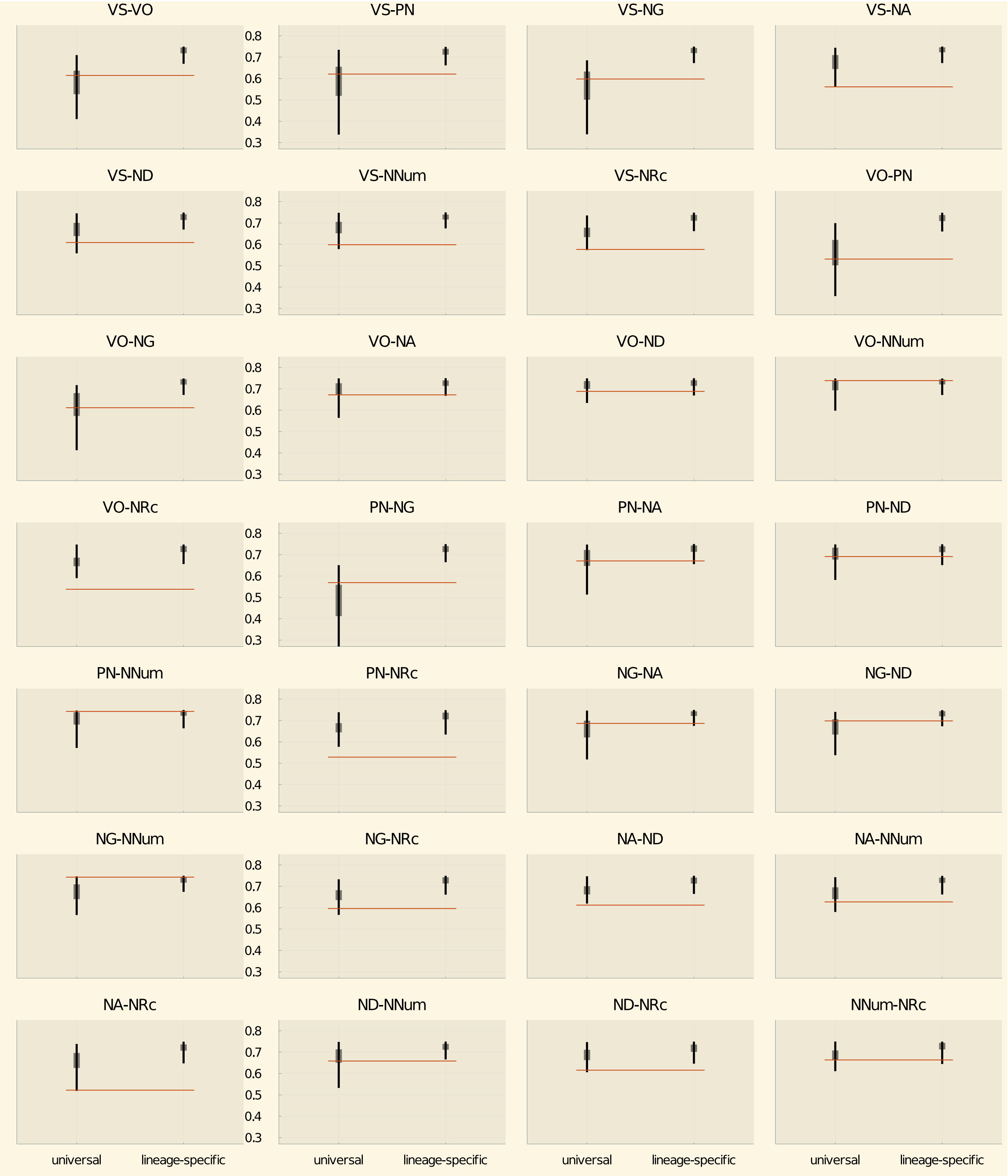

Figure S1. Posterior predictive simulations: total variance. Horizontal lines indicate the empirical value. The thick vertical lines show the 50% highest-density intervals and the thin lines the 95% highest-density intervals of the posterior predictive distributions.

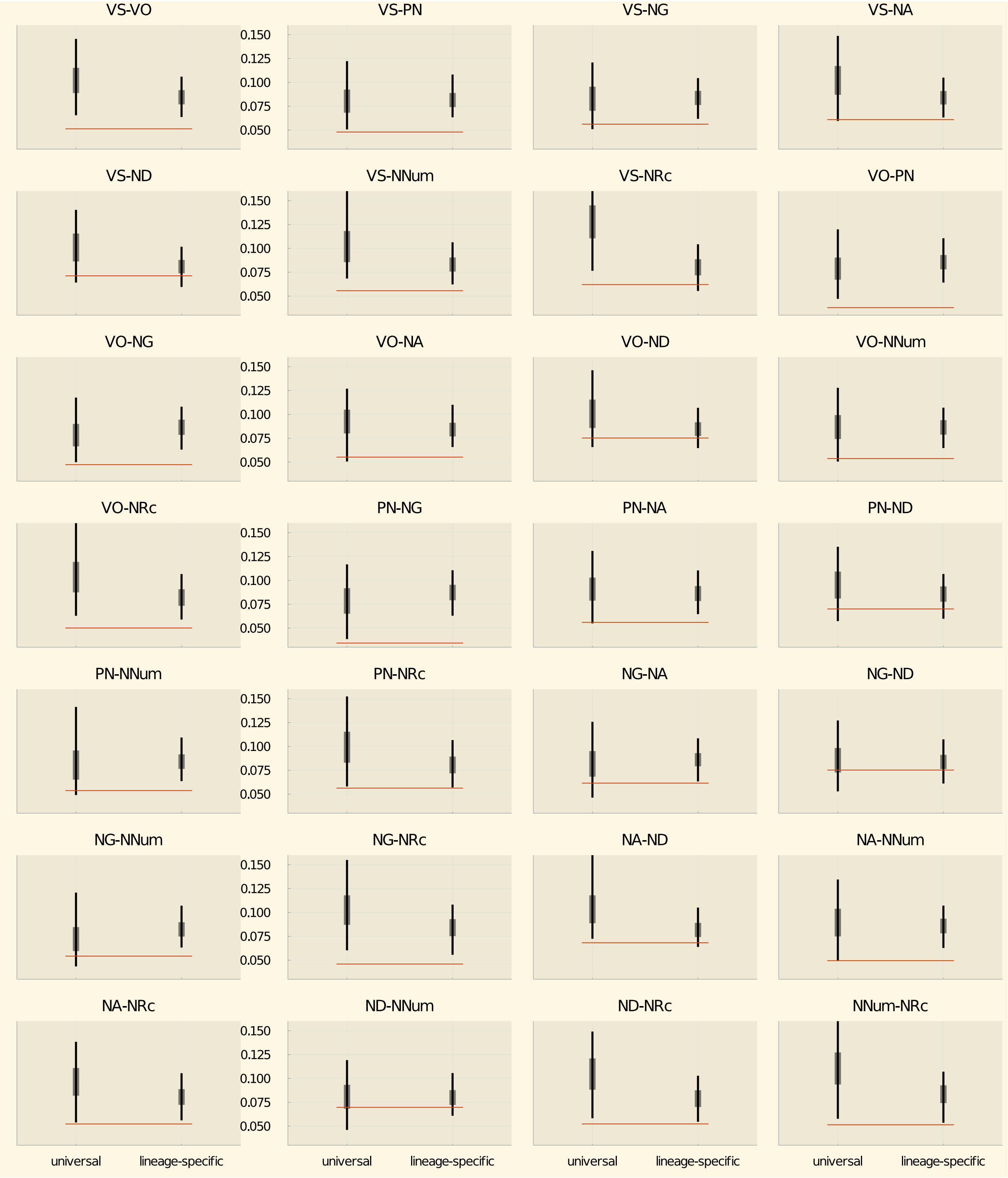

Figure S2. Posterior predictive simulations: lineage-wise variance. Horizontal lines indicate the empirical value. The thick vertical lines show the 50% highest-density intervals and the thin lines the 95% highest-density intervals of the posterior predictive distributions.

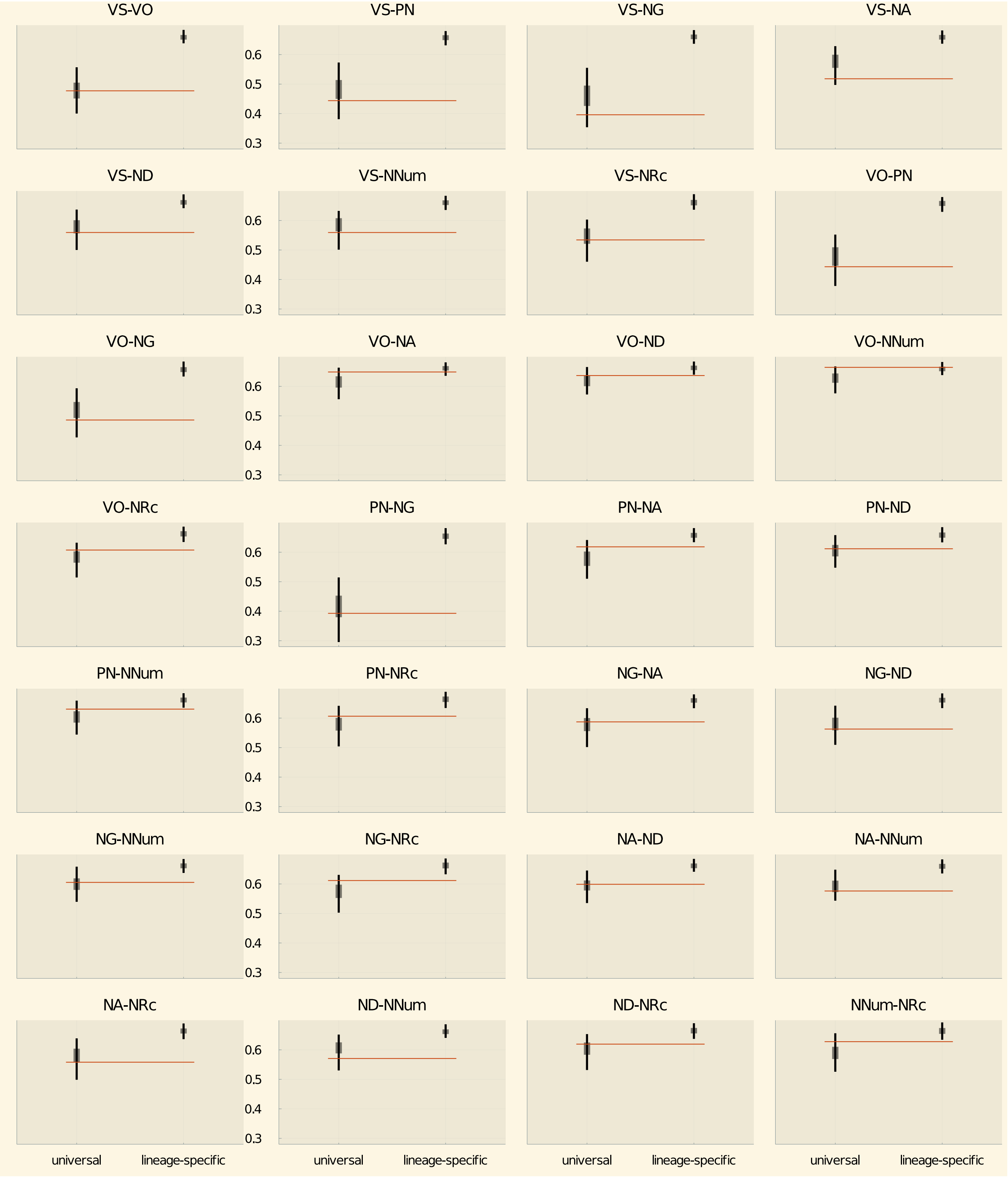

Figure S3. Posterior predictive simulations: cross-lineage variance. Horizontal lines indicate the empirical value. The thick vertical lines show the 50% highest-density intervals and the thin lines the 95% highest-density intervals of the posterior predictive distributions.

feature pair	empirical	universal model (HPD)	lineage-specific model (HPD)
VS-VO	0.477	**(0.400, 0.557)**	(0.638, 0.684)
VS-PN	0.444	**(0.381, 0.573)**	(0.631, 0.680)
VS-NG	0.396	**(0.354, 0.555)**	(0.637, 0.683)
VS-NA	0.518	**(0.497, 0.629)**	(0.637, 0.682)
VS-ND	0.559	**(0.500, 0.637)**	(0.642, 0.688)
VS-NNum	0.559	**(0.501, 0.632)**	(0.636, 0.684)
VS-NRc	0.534	**(0.460, 0.603)**	(0.636, 0.689)
VO-PN	0.443	**(0.378, 0.552)**	(0.629, 0.679)
VO-NG	0.486	**(0.427, 0.593)**	(0.633, 0.684)
VO-NA	0.649	**(0.556, 0.663)**	**(0.636, 0.681)**
VO-ND	0.637	**(0.572, 0.666)**	(0.640, 0.684)
VO-NNum	0.664	**(0.576, 0.668)**	**(0.638, 0.683)**
VO-NRc	0.607	**(0.514, 0.632)**	(0.635, 0.687)
PN-NG	0.393	**(0.296, 0.515)**	(0.627, 0.682)
PN-NA	0.618	**(0.510, 0.641)**	(0.634, 0.682)
PN-ND	0.612	**(0.548, 0.658)**	(0.633, 0.685)
PN-NNum	0.630	**(0.544, 0.659)**	(0.635, 0.684)
PN-NRc	0.606	**(0.504, 0.642)**	(0.634, 0.689)
NG-NA	0.587	**(0.502, 0.634)**	(0.633, 0.680)
NG-ND	0.563	**(0.509, 0.642)**	(0.634, 0.684)
NG-NNum	0.605	**(0.540, 0.659)**	(0.637, 0.685)
NG-NRc	0.612	**(0.503, 0.631)**	(0.633, 0.686)
NA-ND	0.599	**(0.535, 0.646)**	(0.641, 0.685)
NA-NNum	0.576	**(0.543, 0.648)**	(0.636, 0.683)
NA-NRc	0.558	**(0.499, 0.639)**	(0.636, 0.689)
ND-NNum	0.571	**(0.530, 0.652)**	(0.641, 0.687)
ND-NRc	0.619	**(0.532, 0.653)**	(0.637, 0.690)
NNum-NRc	0.628	**(0.526, 0.656)**	(0.634, 0.693)

Table S3. Posterior predictive simulation: Cross-lineage variance. Intervals are shown in bold if they include the empirical value.

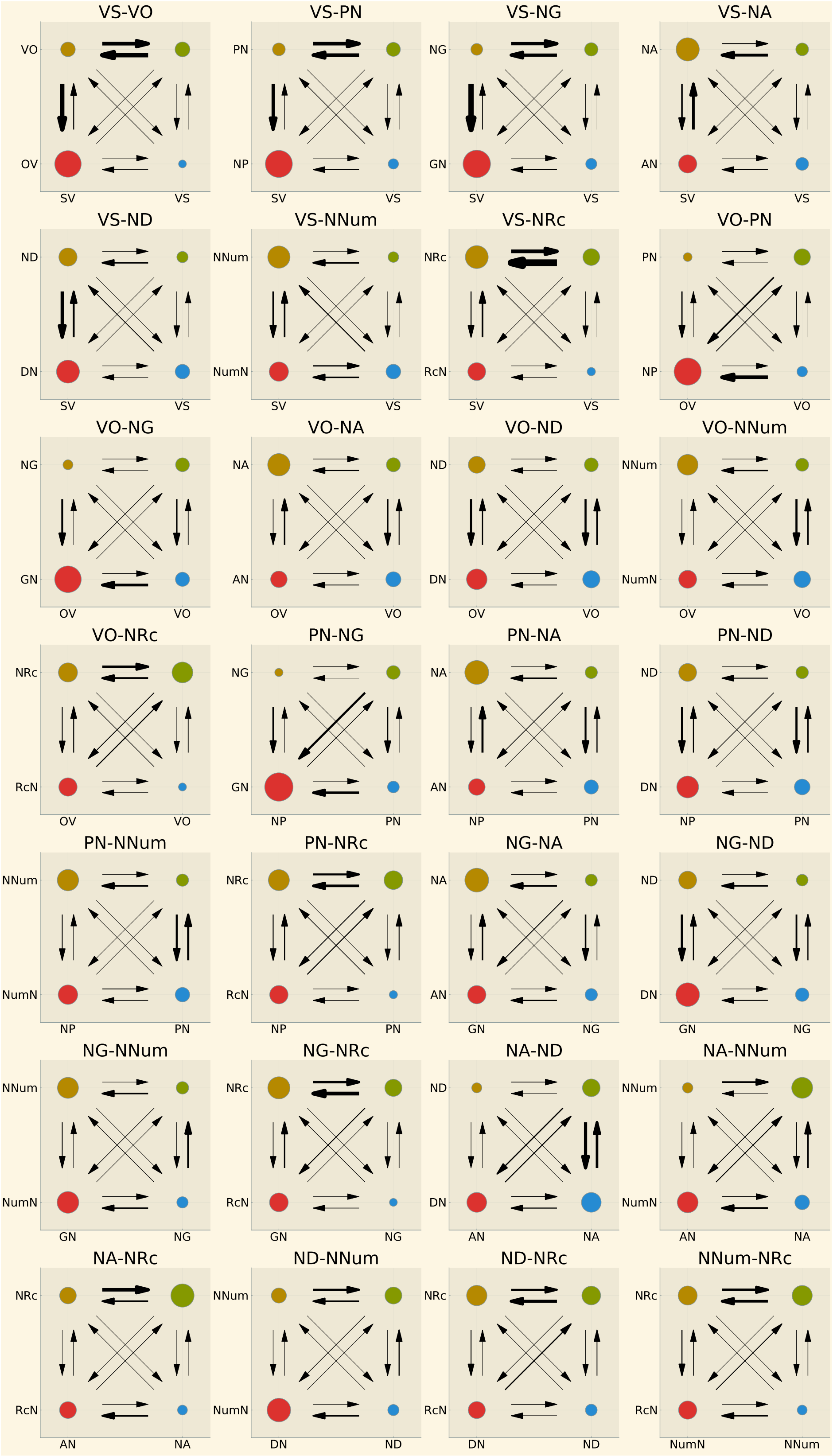

Figure S4. Fitted CTMC parameters. Thickness of the arrows represents the median posterior values of the corresponding rates, and area of the circles is proportional to the median equilibrium distributions of the corresponding states.
